# Identification of MiR-211-5p as a tumor suppressor by targeting ACSL4 in Hepatocellular Carcinoma

**DOI:** 10.1186/s12967-020-02494-7

**Published:** 2020-08-28

**Authors:** Xia Qin, Jian Zhang, Yu Lin, Xue-ming Sun, Jia-ning Zhang, Zhi-qiang Cheng

**Affiliations:** 1 Department of General Surgery, Qilu Hospital of Shandong University, No. 107, western culture road, Jinan, China; 2 The Graduate School of Second Military Medical University, Shanghai, China; 3 Department of Thoracic Surgery, Shandong Cancer Hospital and Institute, Shandong First Medical University and Shandong Academy of Medical Sciences, Jinan, China; 4 School of Medicine and Life Sciences, University of Jinan, Shandong Academy of Medical Sciences, Jinan, China; 5 The Graduate School of Fujian Medical University, Fuzhou, China; 6 Department of Neonatology, Yidu Central Hospital of Weifang, No. 4138, Linglongshan Road, Qingzhou, China; 7 The Third Department of Hepatic Surgery, Eastern Hepatobiliary Surgery Hospital, Shanghai, China

**Keywords:** MicroRNA-211-5p, ACSL4, Hepatocellular carcinoma, Tumorigenesis, Migration, Invasion

## Abstract

**Background:**

Liver cancer is among the most common malignancy worldwide. Hepatocellular carcinoma (HCC), the principal histological subtype of liver cancer, is globally the third most common cause of cancer-related mortality. The high rates of recurrence and metastasis contribute to the poor prognosis of HCC patients. In recent years, increasing evidence has shown that microRNAs (miRNAs) are involved in the tumorigenesis, progression, and prognosis of HCC.

**Methods:**

To screen for key candidate miRNAs in HCC, three microarray datasets were downloaded from Gene Expression Omnibus (GEO). The sole common differentially expressed miRNA (DEmiR) observed in the above three datasets using a Venn diagram was microRNA-211-5p (miR-211-5p). The expression of miR-211-5p from HCC tissues was measured in several HCC cell lines. Additionally, using Kaplan–Meier plots, the potential prognostic value of miR-211-5p in HCC was analyzed. Cell counting kit-8 (CCK-8) and transwell assays examined the ability of miR-211-5p to induce cell proliferation, migration, and invasion in HCC cultures. The interaction of miR-211-5p and Acyl-CoA Synthetase Long Chain Family Member 4 (ACSL4) was assessed both theoretically and using a luciferase reporter assay. Finally, the ability of miR-211-5p to modulate tumorigenesis in HCC in vivo was assessed after establishing a xenograft model.

**Results:**

qRT-PCR demonstrated that the relative expression of miR-211-5p was considerably down-regulated in HCC tissues and cell lines compared with normal tissue. Kaplan–Meier plots indicated that HCC patients with decreased expression of miR-211-5p had poor overall survival. Upregulation of miR-211-5p in vitro consistently suppressed cell proliferation, migration, and invasion. In contrast, enhanced expression of ACSL4 promoted a malignant phenotype in HCC cells. Importantly, we discovered that ACSL4 was a direct downstream target of miR-211-5p in HCC, and that miR-211-5p suppressed the malignant phenotype by inhibition of ACSL4 expression. Furthermore, miR-211-5p overexpression impaired tumorigenesis and growth of HCC in vivo.

**Conclusions:**

Targeting miR-211-5p and the downstream gene ACSL4 will possibly provide novel insight and represents a promising approach to future therapy of HCC patients.

## Background

Primary liver cancer has become the seventh most common cancer and the second most common cause of cancer mortality worldwide, responsible for 819 000 deaths per year [1]. According to data from the National Central Cancer Registry of China in 2015, primary liver cancer has become among the most malignant tumors causing the fourth-highest morbidity and third-highest mortality [2]. Hepatocellular carcinoma (HCC) accounts for up to 90% of all primary hepatic malignancies, with poor prognosis in regions with high prevalence of hepatitis B and C [3]. The 5-year survival rate of HCC in China remains less than 5% in spite of considerable therapeutic advances [2]. Although many studies have clarified the molecular mechanisms and signal pathways closely related to the development of HCC[4–6], the precise mechanisms for the occurrence and progression of HCC remain incompletely elucidated and require further investigation.

MicroRNAs (miRNAs), a class of non-coding RNAs with a length of 18 to 25 nucleotides, can regulate numerous cellular processes such as proliferation, apoptosis, cell cycle progression, differentiation, and DNA repair by suppressing translation of its target mRNA or by the promotion of mRNA degradation [7, 8]. Furthermore, extensive research has found that over-expression or under-expression of a particular miRNA can regulate tumor cell proliferation, invasion and metastasis by modulating the expression of its target genes [9–11]. Therefore, identification of abnormally-expressed miRNAs and their downstream target genes in HCC enhance our understanding of the potential pathogenesis of HCC and represents a promising strategy for the diagnosis, treatment, and prognosis of HCC.

In recent years, with the development of gene expression profiling chips and the emergence of next-generation sequencing, considerable quantities of HCC expression profiling data have emerged, providing the basis for the comprehensive study of differentially expressed genes in HCC and their biological function. In the present study, three datasets of gene expression profiles of HCC and normal liver tissues were downloaded from the Gene Expression Omnibus (GEO) database and the abnormally expressed miRNAs and target genes explored in terms of their occurrence and progression in patients with HCC. The results may contribute towards the identification of a novel therapeutic target for HCC.

## Materials and methods

### Transcriptional expression profile

Three datasets of gene expression profiles of hepatocellular carcinoma and normal liver tissues (GSE85677, GSE112264, and GSE108724) were obtained from the free public database, NCBI-GEO. The data for GSE85677 was downloaded from GPL21263 (3D-Gene Human miRNA V21_1.0.0) and included 42 tissues from HCC patients. The data for GSE112264 was also downloaded from GPL21263 (3D-Gene Human miRNA V21_1.0.0) and consisted of 91 male samples, including 50 HCC tissues and 41 non-cancer controls. The data for GSE108724 was downloaded from GPL20712 (Agilent-070156 Human miRNA [miRNA version]) and included 7 pairs of HCC and matched adjacent tumor-free tissues.

### Data preprocessing and standardization of DemiRs

The Datasets were microarray studies, in which gene expression levels were measured using 3D-Gene Human miRNA V21_1.0.0 (GSE85677 and GSE112264), or Agilent-070156 Human miRNA (GSE108724), due to designs used by different manufacturers. Corresponding annotation information was provided by the GEO database, while a number of probes were designed as controls without gene annotation information.

The data for each dataset were downloaded from the GEO database. A robust multi-array average (RMA) algorithm was used to perform background adjustment, quantile normalization, and final summarization of oligonucleotides per transcript using the median polish algorithm. The k-nearest neighbor (KNN) algorithm was then used using the Impute package within Bioconductor software to impute any missing values. Batch effects in the expression data were adjusted using an empirical Bayes method using the R package sva, in accordance with previous studies. Data were analyzed using the R (version 3.6.1) programming environment and R Bioconductor packages.

### Patients and tissue samples

A total of 396 HCC patients that had been consecutively recruited with available miRNA-sequence data were downloaded from the Cancer Genome Atlas (TCGA) database [12]. The miRNA expression profiles had been recorded experimentally using an Illumina miRNA Sequencing platform by the University of North Carolina TCGA Genome Characterization Center. X-tile software was used to select a threshold miRNA expression value for miR-211-5p, which was used to divide participants into one of two groups.

Thirty pairs of HCC tissues and matched adjacent tumor-free tissues were obtained from patients with HCC who had undergone surgical resection at the Qilu Hospital of Shandong University (Jinan, China). Tissues were frozen in liquid nitrogen and used for qRT-PCR. Informed consent was obtained from all patients The study protocol was approved by the institutional review board of the Qilu Hospital of Shandong University.

### Cell culture and transfection

Human hepatic cells (QSG-7701) and HCC cell lines (Huh-7, SK Hep-1, HepG2, and LM3) were purchased from the American Type Culture Collection (ATCC, Manassas, VA, USA). Huh-7, SK Hep-1, HepG2, and LM3 were cultured in Dulbecco’s modified Eagle’s medium (DMEM; Gibco, Shanghai, China) supplemented with 10% fetal bovine serum (FBS, Gibco, Shanghai, China) and 1% penicillin/streptomycin. QSG-7701 cells were cultured in RPMI 1640 medium supplemented with 10% FBS and 1% penicillin/streptomycin. All cells were cultured in a humidified atmosphere containing 5% CO_2_ at 37 °C.

For cell transfection, a miR-211-5p mimic (miR10000268-1-5), miR-211-5p inhibitor (miR20000268-1-5), and negative control (NC, miR1N0000002-1-5) were purchased from Ribobio Co. Ltd (Guangzhou, China). Small interfering RNAs (siRNAs) specific for ACSL4 (siACSL4-1 and siACSL4-2) were synthesized. The sequences were as follows: siACSL4-1: sense 5′-UUCCGAUUCGUAUCACGGUUU-3′ and antisense 5′-GACACUUGGCCAGCAUACCUU-3′; siACSL4-2: sense 5′-GAGGCUUCCUAUCUGAUUATT-3′ and antisense 5′-UAAUCAGAUAGGAAGCCUCTT-3′. A corresponding scramble siRNA was used as a negative control (Ribobio, China). SK Hep-1 and LM3 cells were transfected with 50 nM miRNAs, 3 µmol/l siRNAs, and 1 µg/µl ACSL4 overexpression plasmid (pcDNA3.1-ACSL4, obtained from Shanghai GeneChem Co., Ltd.) using Lipofectamine™ 3000 reagent (Invitrogen), in accordance with protocols from Invitrogen. Cells were transfected for 48 h then the transfection examined using qRT-PCR.

### Total RNA extraction, target prediction, and quantitative real-time PCR analysis

All RNAs were extracted from cells and clinical samples using Trizol reagent (Thermofisher Scientific) in accordance with instructions provided by Thermofisher. The extracted RNA was reverse transcribed into cDNA using a PrimeScript® RT reagent kit (ThermoFisher, China), then qRT-PCR was performed using a LightCycler® 96 (Roche Diagnostics, Germany) using SYBR Green (Roche Diagnostics, Germany). The expression of miR-211-5p was quantified using an miRNeasy kit (Qiagen) and a miScript SYBR Green PCR kit (Qiagen). RNU6 (miRNA) served as an endogenous control. The sequences of primers for miR-211-5p were: forward: 5′-GATGCTGTAATGGATGATATGA-3′, and reverse: 5′-ATTGGAACGATACAGAGAAGATT-3′. Sequences of primers for RNU6 were: forward: 5′-CTCGCTTCGGCAGCACA-3′ and reverse: 5′-ACGCTTCACGAATTTGCGT-3′. ACSL4 primers were: forward: 5′-AACCCAGAAAACTTGGGCATT-3′, and reverse: 5′-GTCGGCCAGTAGAACCACT-3′. GAPDH was used as an internal control for normalization of mRNA expression levels. The GAPDH primer sequences were: forward: 5′- ACAACTTTGGTATCGTGGAAGG-3′, and reverse: 5′-GCCATCACGCCACAGTTTC-3′. All results were replicated in triplicate. The 2^−ΔΔCt^ method was used to calculate relative gene expression levels.

### Target prediction

The targets of miRNAs were predicted using the online biological database TargetScan (https://www.targetscan.org/).

### Luciferase reporter assay

Wild-type (WT) or mutant (MUT) 3′-UTRs of ACSL4 were used to predict the interaction between miR-211-5p and the 3′-UTRs of ACSL4. Lipofectamine 3000 reagent (Invitrogen) was used to co-transfect the two forms of 3′-UTR or of ACSL4 and miR-211-5p mimic into LM3 and SK Hep-1 cells, which were inserted into the pGL3 Basic vector (Promega, Madison, WI, USA) in 96-well plates. Forty eight hours after co-transfection, cells were harvested for additional luciferase analysis using a Dual-Luciferase® Reporter Assay kit (Promega, Madison, WI, USA).

### Cell viability assay

Cells were seeded into the wells of a 96-well plate at a density of 1 × 10^4^ cells/well. After culturing in an incubator for 24 h, 48 h, 72 h, and 96 h respectively, 10 μl CCK8 reagent (Beyotime) were added to each well and incubated for 30 min, in accordance with instructions from Beyotime. The absorbance values at a wavelength of 450 nm were recorded using a BioTek 312e microplate reader (BioTek Instruments, Winooski, VT).

### Western blots

To analyze Western blots, cells were collected and lysed using RIPA buffer (50 mM Tris, pH 7.4, 150 mM NaCl, 1% TritonX-100, 1% sodium deoxycholate, 0.1% SDS, 2 mM sodium pyrophosphate, 25 mM β-glycerophosphate, 1 mM EDTA, 1 mM Na3VO4, and 0.5 μg/mL leupeptin). The lysates were then separated by SDS polyacrylamide gel electrophoresis (SDS-PAGE), and electrophoretically transferred to nitrocellulose (NC) membranes (GE Healthcare Life Science). The membranes were blocked with 5% nonfat milk in PBST for 1 h, then incubated overnight at 4 °C with an appropriate antibody. Secondary antibodies against rabbit or mouse were diluted to 1:5000, incubated with each membrane for 1 h then visualized by chemiluminescence. Primary antibodies were: anti-ACSL4 (Abcam, ab155282), and anti-Actin (Sigma-Aldrich, Cat# A3853).

### Transwell invasion and migration assay

A 24-well transwell chamber (Greiner Bio-one, Switzerland) with or without Matrigel (BD Bioscience, USA) was used to perform transwell invasion or migration assays. A total of 2 × 10^5^ cells were suspended in 0.2 mL of serum-free medium and added to the chamber. Medium supplemented with 10% FBS (0.5 mL) was added to the lower compartment as a chemical attractant. After incubating for 48 h at 37 °C, cells on the upper surface of the membrane were carefully removed with a cotton swab, and cells on the lower surface fixed with 100% methanol then stained with 0.5% crystal violet. Five random fields of magnification at 200× were selected for each insert, and the number of cells counted using an optical microscope (Olympus, Japan).

### Xenograft experiments

LM3 cells transfected with WT and miR-211-5p mimic were used to establish a xenograft model in vivo. All animal experiments were performed in accordance with National Institutes of Health guidelines and approved by the animal care and use committee of Qilu Hospital of Shandong University. Twenty male BALB/c nude mice (5 weeksold) were randomly divided into two groups, then injected subcutaneously into the right axilla with 5 × 10^6^ WT LM3 cells or those transfected with miR-211-5p mimic, depending on the grouping of the mouse, each suspended in 0.1 mL PBS plus 0.1 mL Matrigel (BD Bioscience, USA). The body weight and tumor volume of each mouse were measured every 5 days. One month after subcutaneous injection, all mice were sacrificed and the weight of the tumor measured.

### Immunohistochemistry (IHC) and scoring

LM3 xenograft tumors were stained for ACSL4 using IHC, in accordance with standard protocols. Briefly, paraffin sections (4 μm) were dewaxed and antigens retrieved by heat treatment in Tris/EDTA buffer, pH 9. After blocking with 1% bovine serum albumin (BSA) in PBST, tissue sections were incubated with anti-ACSL4 antibody diluted 1:200, overnight at 4 °C. A secondary polyclonal-horseradish peroxidase (HRP) conjugated anti-rabbit IgG antibody (Abcam, China) was incubated at room temperature for 1 h. The immunostained sections were reviewed in a double-blind manner then scored by two experienced pathologists. Using the H-score method, the staining intensity and percentage of positive cells were evaluated by semi-quantitative results. Five visual fields (× 200) were observed from each section, and the total number and staining intensity of the cells recorded for each field. Positive staining intensity: 0 = colorless; 1 = light yellow; 2 = medium brown; 3 = dark brown. An H-score was calculated using the following formula: (% of cells stained at intensity category 1 × 1) + (% of cells stained at intensity category 2 × 2) + (% of cells stained at intensity category 3 × 3). H-scores varied from 0 to 300 where 300 represented 100% of cells that were strongly stained (+ + +).

### Statistical analysis

GraphPad Prism 8.0 software (GraphPad) and the R (v 3.6.1) programming environment were used to conduct statistical analysis. The results of in vitro experiments included at least 3 independent observations. There were at least 5 mice in each group for the xenograft experiments. All measurement data represent means ± standard deviation (SD). Differences in data between groups were analyzed using a Student’s t-test or one-way analysis of variance (ANOVA). P-values < 0.05 were considered statistically significant.

## Results

### Abnormal expression and prognostic values of miR-211-5p in hepatocellular carcinoma

**Fig. 1:**
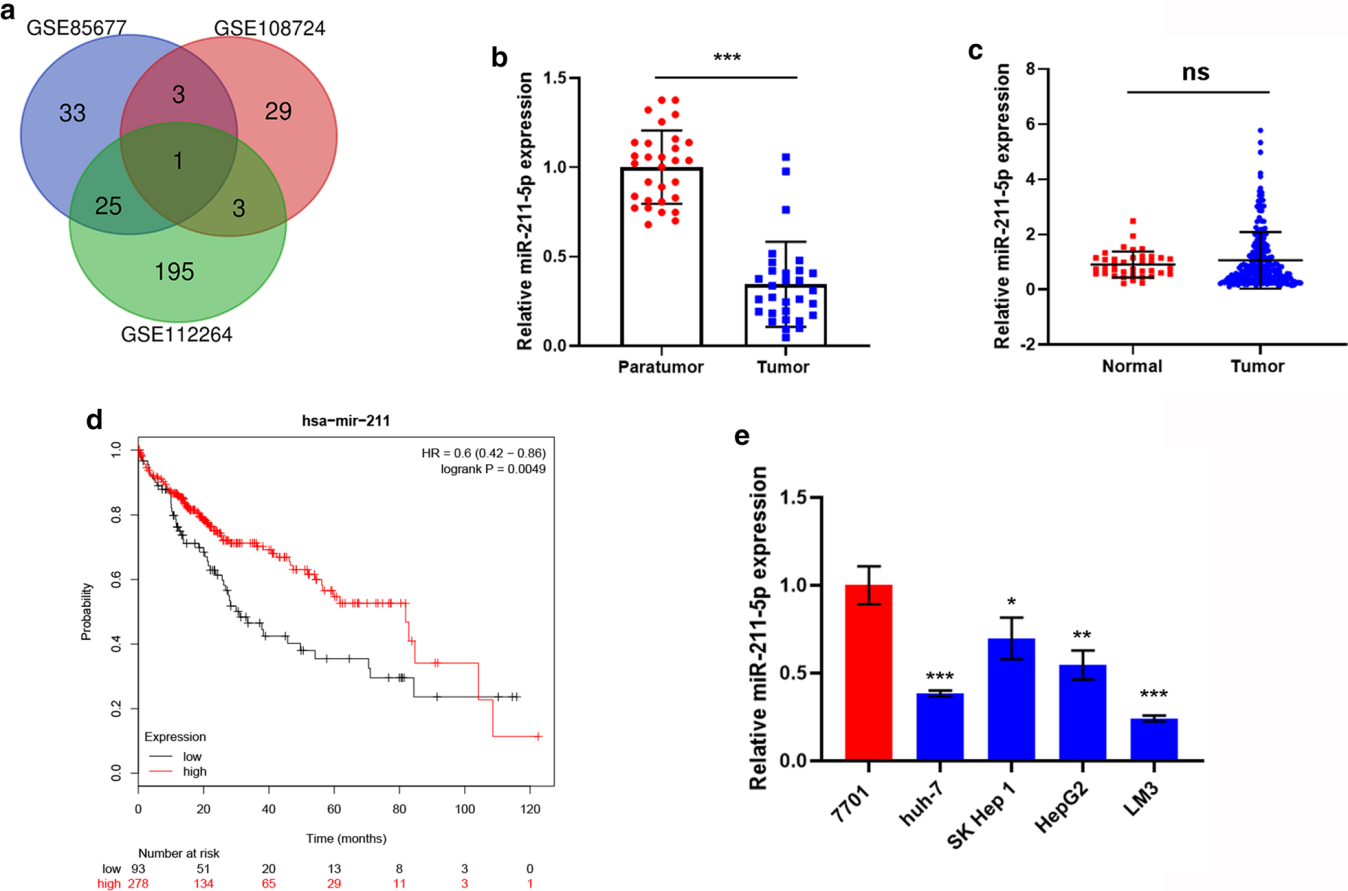
Abnormal expression and prognostic value of miR-211-5p in HCC. **a** The overlap miRNA among the three datasets were displayed in Venn diagram and only the hsa-miR-211-5p was the common. **b** Comparison of miR-211-5p expression between 30 pairs of HCC and matched adjacent tumor-free tissues from patients in clinical. **c** Comparison of miR-211-5p expression between HCC tissues and normal liver tissues based on TCGA cohorts. **d** The overall survival analysis of miR-211-5p in HCC through TCGA cohorts based on online database Kaplan–Meier Plotter. **e** The relative miRNA expression level of miR-211-5p in 4 HCC cell lines (Huh-7, SK Hep-1, HepG2, and LM3) compared with normal liver cells (QSG-7701). *p < 0.05, **p < 0.01, ***p < 0.001

The three GEO datasets, GSE85677, GSE112264, and GSE108724 included data from 99 individual HCC tissues and 48 normal liver tissues. After normalization and censoring of the miRNA microarray information, it was established that the degree of differential expression of DEmiRs was significant, based on the analysis and statistical parameters of the data processing steps. Overlap of the three datasets was displayed using a Venn diagram, from which we found that only hsa-miR-211-5p was common to all three (Fig. 1a). Although miR-211-5p expression in liver cancer samples was not significantly different from adjacent normal samples based on TCGA cohorts, it was considerably down-regulated in 30 pairs of HCC tissues compared with matched adjacent tumor-free tissues from patients in clinic or real-world cohorts (Fig. 1b, c). Additionally, we analyzed the potential prognostic value of miR-211-5p in HCC using online Kaplan–Meier Plotter database, which indicated that HCC patients with a decreased expression of miR-211-5p had poor overall survival (logrank P = 0.0049, Fig. 1d). In addition, the expression of miR-211-5p was measured in several HCC cell lines. As shown in Fig. 1e, consistent with clinical data, the relative expression level of miR-211-5p in HCC cells was significantly lower than in QSG-7701 normal liver cells. Notably, SK Hep-1 and LM3 cells exhibited the greatest and lowest miR-211-5p expression, respectively, and thus were selected for further experimental validation.

### Effects of miR-211-5p on proliferation, migration, and invasion of HCC cells in vitro

**Fig. 2:**
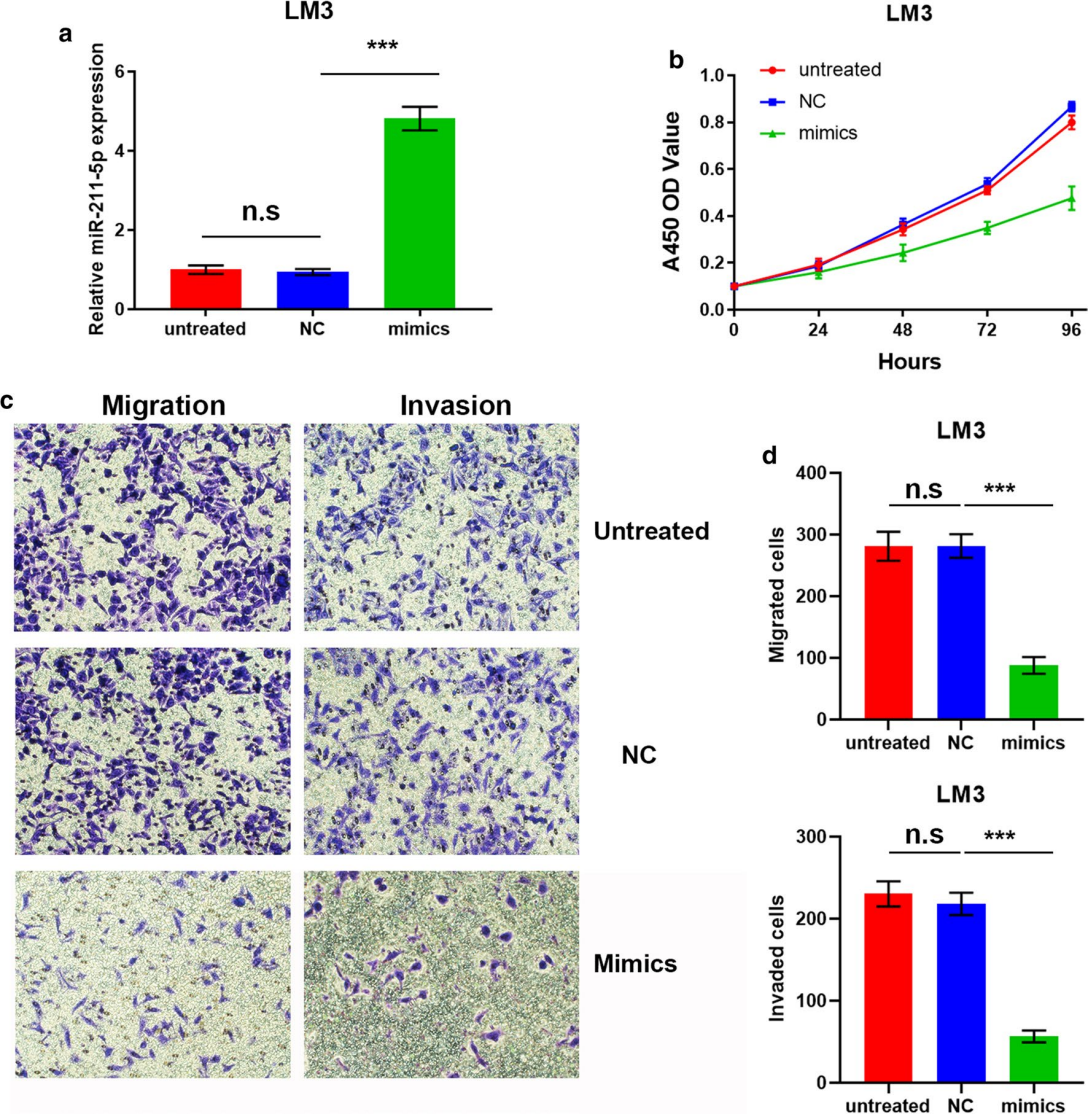
Upregulation of miR-211-5p reduces proliferation, migration, and invasion of LM3 cells. **a** LM3 cells were transfected with miR-211-5p mimics and corresponding miR-NC. The effect of transfection was tested by qRT-PCR. **b** CCK8 assay was performed to examine the proliferative ability of untreated LM3 cells, LM3 transfected with miR-NC, and LM3 transfected with mimics. **c** Representative images (×200) from migrated and invaded LM3 cells of untreated group, NC group, and mimics group. As expected, the number of migrated cells was more than that of invasive cells in each group. **d** Quantification analysis of results from **c**. *p < 0.05, **p < 0.01, ***p < 0.001

**Fig. 3:**
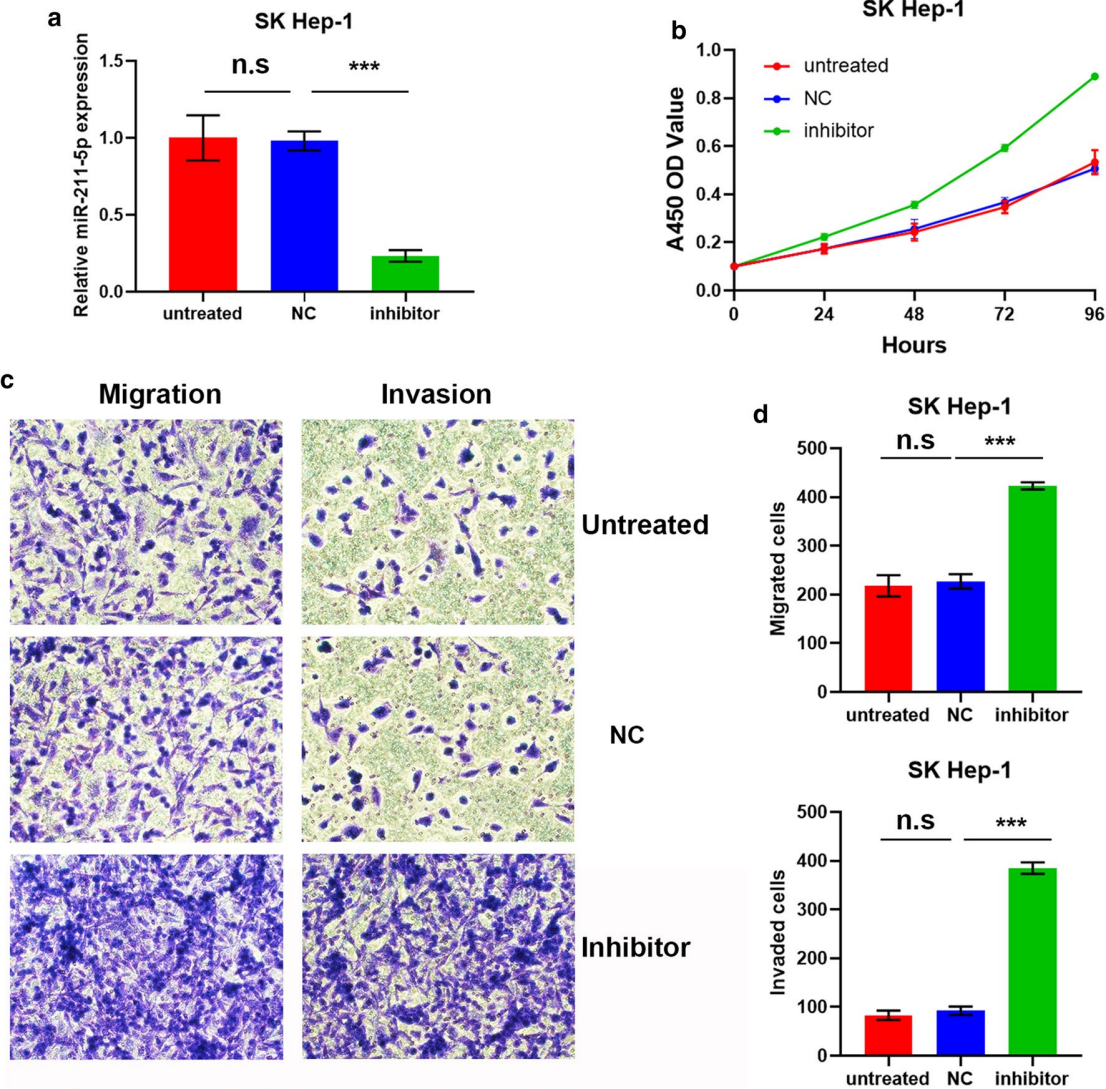
miR-211-5p silencing promotes proliferation, migration, and invasion of SK Hep-1 cells. **a** SK Hep-1 cells were transfected with miR-211-5p inhibitor and corresponding miR-NC. The effect of transfection was tested by qRT-PCR. **b** CCK8 assay was performed to examine the proliferative ability of untreated SK Hep-1 cells, SK Hep-1 transfected with miR-NC, and SK Hep-1 transfected with inhibitor. **c** Representative images (×200) from migrated and invaded SK Hep-1 cells of untreated group, NC group, and inhibitor group. **d** Quantification analysis of results from **c**. *p < 0.05, **p < 0.01, ***p < 0.001

To explore whether miR-211-5p played a role in the progression of HCC, miR-211-5p mimic and the corresponding miRNA negative control (miR-NC) were transfected into LM3 cells, and used in gain-of-function experiments. The qRT-PCR results demonstrated that miR-211-5p mimic significantly enhanced the expression levels of miR-211-5p (Fig. 2a). Results of the CCK8 assay indicated that overexpression of miR-211-5p significantly suppressed the proliferative capability of LM3 cells (Fig. 2b). Furthermore, as shown in Fig. 2c, d, the transfection of miR-211-5p mimic into LM3 cells clearly reduced the migration and invasion of LM3 cells, as observed in transwell assays. Loss-of-function experiments were conducted by transfecting miR-211-5p inhibitor and the corresponding miR-NC into SK Hep-1 cells. As expected, miR-211-5p inhibitor significantly inhibited the expression of miR-211-5p (Fig. 3a). The proliferative capability of SK Hep-1 cells was considerably elevated after transfection of miR-211-5p inhibitor (Fig. 3b). Consistent with the results above, miR-211-5p silencing significantly promoted the migration and invasion of SK Hep-1 cells (Fig. 3c, d).

To identify the comparability between gain-of-function and loss-of-function experiments, LM3 and SK Hep-1 cells were both transfected with miR-211-5p mimic or inhibitor. As shown in Additional file 1: Fig. S1A, the effects of mimic and inhibitor in both LM3 and SK Hep-1 cells were confirmed by qRT-PCR. Furthermore, the expression of miR-211-5p mimic in LM3 cells exceeded that of WT expression levels in SK Hep-1 cells, and that miR-211-5p expression in SK Hep-1 cells following inhibitor treatment decreased to levels comparable with those of WT LM3 cells, confirming the validity of the experimental design.

### MiR-211-5p negatively modulates ACSL4 expression levels

**Fig. 4:**
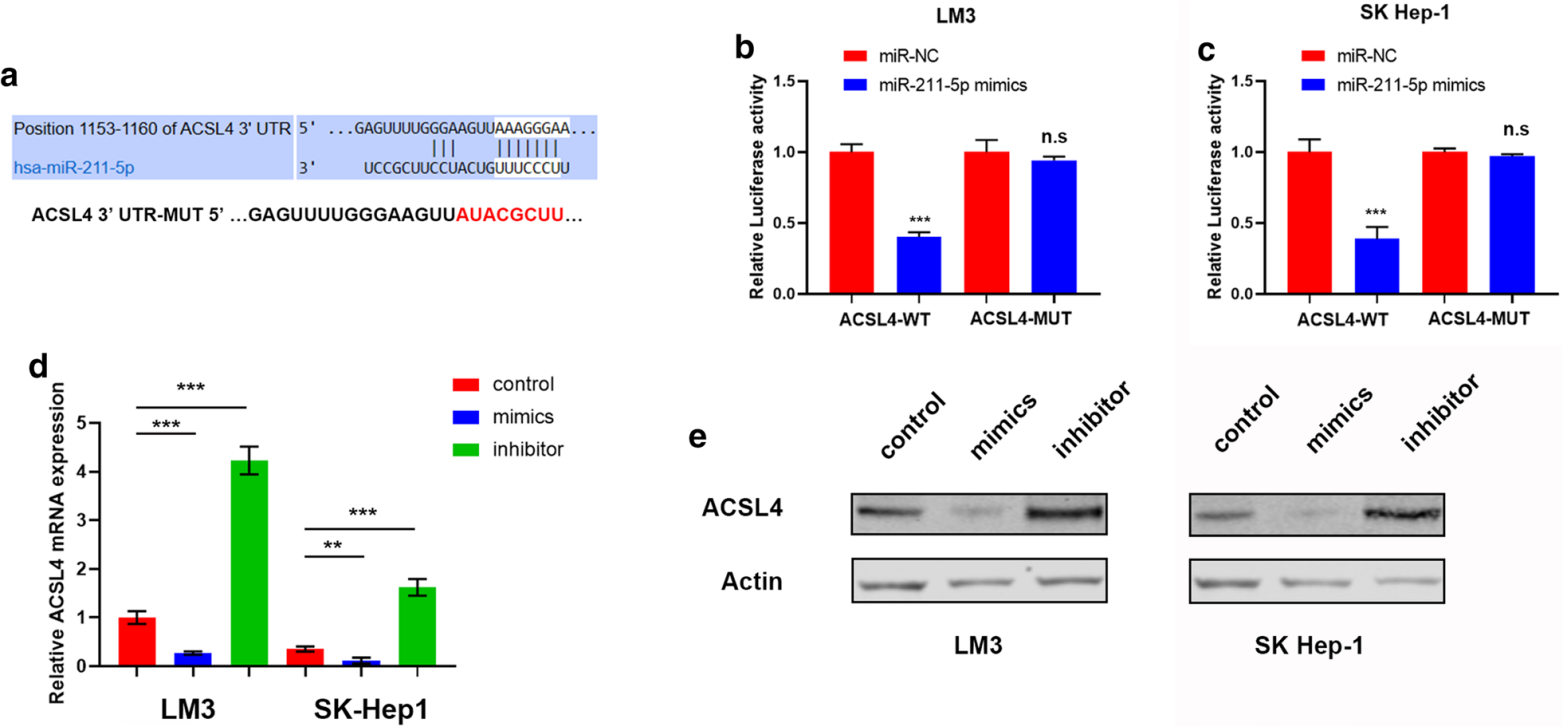
MiR-211-5p binds with 3′-UTR of ACSL4 and negatively modulates ACSL4 expression. **a** The 3′-UTR of ACSL4 contains a putative binding site for miR-211-5p and a mutation of the 3′-UTR of ACSL4 was designed. **b**, **c** The luciferase assay indicated that overexpression of miR-211-5p by mimic significantly reduced the luciferase activity of ACSL4-WT, while this difference could not be observed in the luciferase activity of ACSL4-MUT in LM3 (**b**) and SK Hep-1 (**c**) cells. **d** The results of qRT-PCR showed the relative ACSL4 mRNA expression level of LM3 and SK Hep-1 cells in control group, transfecting miR-211-5p mimics group, and transfecting miR-211-5p inhibitor group. **e** The protein expression level of ACSL4 and β-Actin in LM3 and SK Hep-1 cells. *p < 0.05, **p < 0.01, ***p < 0.001

The underlying molecular mechanisms of the biological function of miR-211-5p in HCC cells was next investigated. Using the TargetScanHuman 7.2 online tool (https://www.targetscan.org), the Acyl-CoA Synthetase Long Chain Family Member 4 (ACSL4) gene was identified as a potential downstream target of miR-211-5p, with a 3′-UTR of ACSL4 containing a putative binding site for miR-211-5p (Fig. 4a). A luciferase assay was then performed to validate this result. A mutation of the 3′-UTR of ACSL4 (ACSL4-MUT, Fig. 4a) was designed. As shown in Fig. 4b, c, overexpression of miR-211-5p by the mimic significantly reduced luciferase activity in ACSL4-WT, while this difference was not observed with ACSL4-MUT, suggesting that the 3′-UTR of ACSL4 binds with miR-211-5p. Furthermore, we also investigated whether miR-211-5p regulated the expression of ACSL4. As shown in Fig. 4d, e, transfection of miR-211-5p mimics or inhibitor into LM3 and SK Hep-1 cells significantly inhibited or promoted, respectively, ACSL4 expression levels. Taken together, these results indicate that ACSL4 is a direct downstream target of miR-211-5p in HCC, and miR-211-5p downregulated ACSL4 expression levels.

### Enhanced expression of ACSL4 promotes the malignant phenotype of HCC cells

ACSL4 was originally identified as having non-specific mutations in X-linked mental retardation [13]. So far, ACSL4 disorders have also been shown to be associated with multiple malignancies [14–16]. Furthermore, ACSL4 has been shown to be overexpressed in HCC with high expression of ACSL4 predicted to have poor prognosis in HCC [17–19]. However, whether ACSL4 plays a role in the growth and metastasis of HCC cells remains largely unknown. Therefore, we first examined the expression levels of ACSL4 in a number of HCC cell lines and QSG-7701 cells. Consistent with previous studies, the mRNA and protein expression of ACSL4 was enhanced in HCC cells compared with QSG-7701 cells (Additional file 2: Figure S2). Surprisingly, the relative expression levels of ACSL4 in these cell lines is the converse of that of miR-211-5p in the same cell lines, as displayed in Fig. 1e. The effect of ACSL4 in the progression of HCC was then explored by transfection of two siRNAs specific for ACSL4 (siACSL4-1 and siACSL4-2) and the ACSL4 overexpression plasmid pcDNA3.1-ACSL4 (ACSL4 OE) into LM3 and SK Hep-1 cells. As shown in Additional file 2: Figure S2, the expression of ACSL4 was silenced in LM3 and SK Hep-1 cells by siACSL4-2 but not by siACSL4-1, and pcDNA3.1-ACSL4 markedly enhanced ACSL4 expression in both cell lines. The proliferation of LM3 and SK Hep-1 cells was considerably elevated in the ACSL4 OE group. Conversely, siACSL4-2 significantly suppressed proliferation in the two cell lines (Additional file 2: Figure S2). Consistently, we observed that ACSL4 overexpression promoted the migration and invasion of LM3 and SK Hep-1 cells, but silencing of ACSL4 by siACSL4-2 markedly suppressed the migration and invasion of the two cell lines (Additional file 2: Figure S2). Briefly, the results demonstrate that the enhanced expression of ACSL4 promoted a malignant phenotype of HCC cells, the converse of the effects of miR-211-5p on the phenotype of HCC.

### MiR-211-5p suppresses proliferation, migration, and invasion of HCC cells by targeting ACSL4

**Fig. 5:**
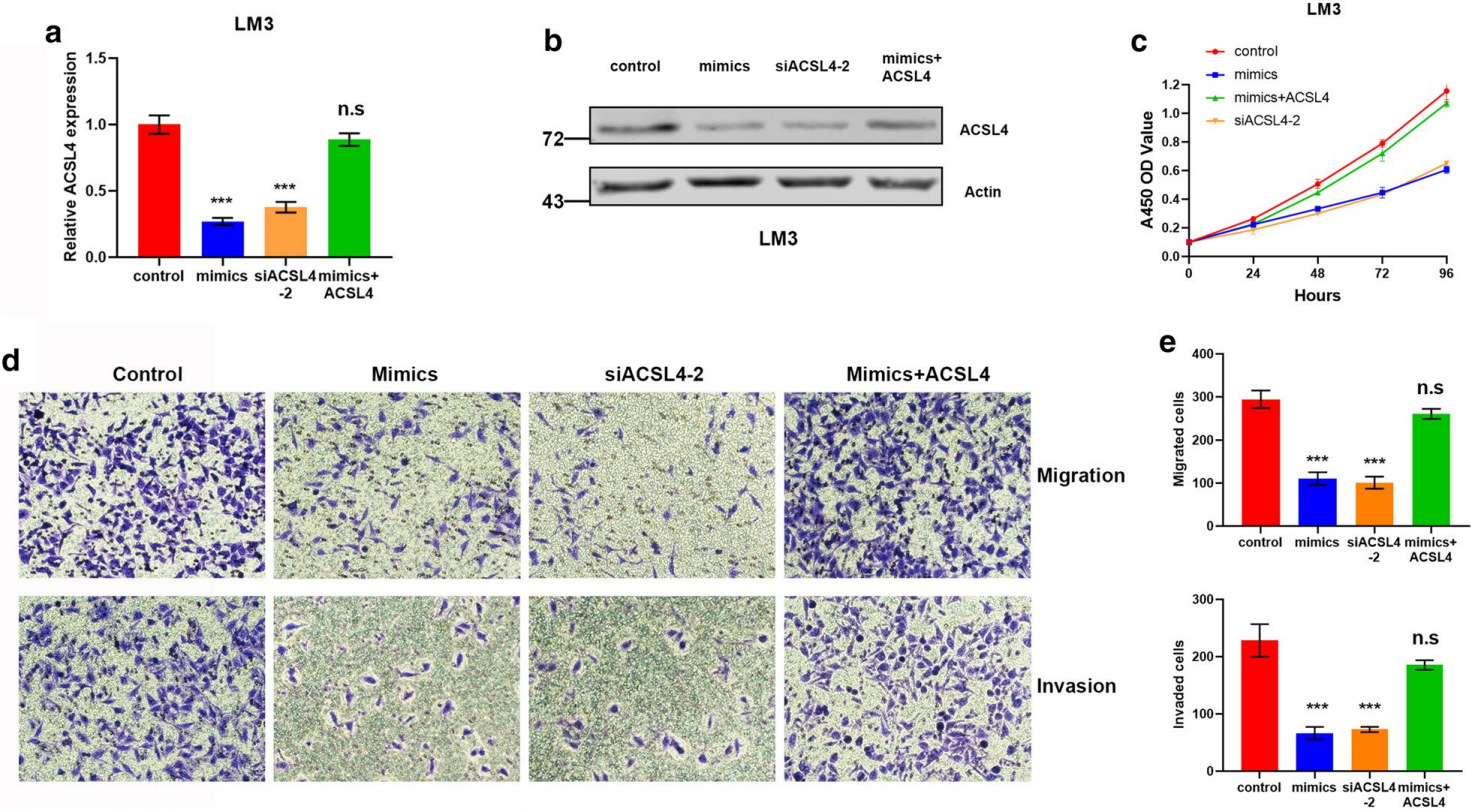
MiR-211-5p suppresses proliferation, migration and invasion of HCC cells by targeting ACSL4. The mRNA (**a**) and protein (**b**) expression level of ACSL4 in negative control group, transfecting miR-211-5p mimics group, transfecting siACSL4-2 group, and transfecting miR-211-5p mimics plus pcDNA3.1-ACSL4 group. **c** The proliferation ability of LM3 cells in negative control group, miR-211-5p mimics group, transfecting siACSL4-2 group, and ACSL4 rescue group. **d** Comparison of migration and invasion of LM3 cells between negative control group, miR-211-5p mimics group, transfecting siACSL4-2 group, and ACSL4 rescue group. **e** Quantification analysis of results from (D). *p < 0.05, **p < 0.01, ***p < 0.001

Because ACSL4 is a direct downstream target of miR-211-5p, and enhanced expression of ACSL4 promotes the proliferation, migration and invasion of HCC cells, we hypothesized that miR-211-5p would suppress the progression of HCC by down-regulating ACSL4. Firstly, miR-211-5p mimic, siACSL4-2, and miR-211-5p mimic plus pcDNA3.1-ACSL4 were transfected into LM3 cells, after which the change in ACSL4 mRNA and protein expression levels were quantified. As shown in Fig. 5a, b significant reduction in ACSL4 expression was detected in the mimic and siACSL4-2 transfection groups, while this expression was rescued to a normal level in the miR-211-5p mimic plus pcDNA3.1-ACSL4 transfection group. A CCK-8 assay was then conducted to determine whether ACSL4 rescued the proliferative capability of HCC cells after miR-211-5p mimic transfection. The results demonstrated that proliferation was substantially attenuated in the miR-211-5p upregulation and siACSL4-2 transfection groups, while the OD values were rescued to normal in the ACSL4 rescue group (Fig. 5c). Likewise, transwell assays indicated that invasion and migration of LM3 cells were clearly attenuated in the miR-211-5p upregulation and siACSL4-2 transfection groups, while cell numbers in the ACSL4 rescue group were rescued to levels comparable with those in the control group (Fig. 5d, e). Therefore, the data demonstrate that miR-211-5p suppressed a malignant phenotype in HCC cells by targeting ACSL4.

### Overexpression of miR-211-5p inhibits ACSL4 expression and growth of xenograft tumors in vivo

**Fig. 6:**
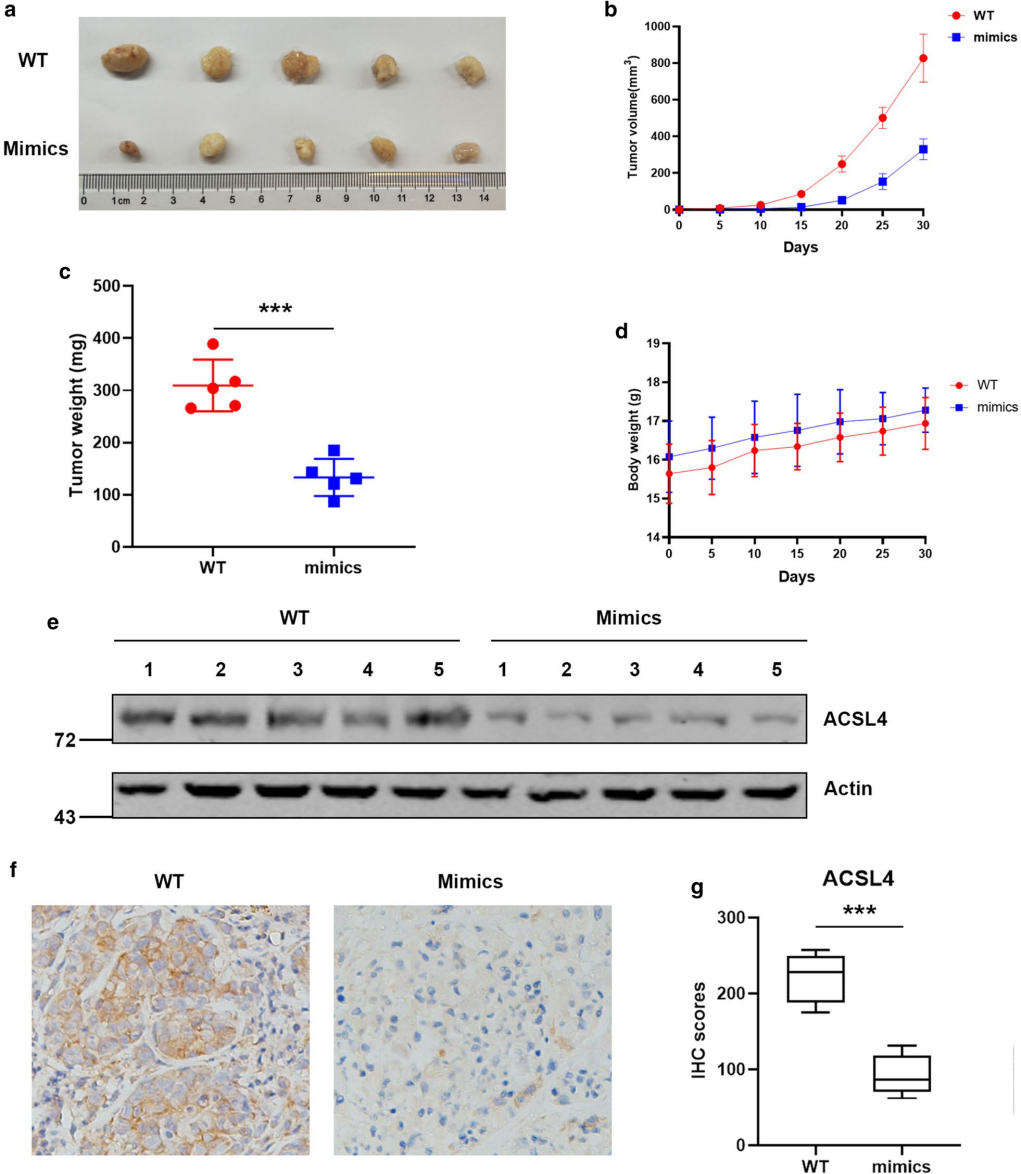
Xenograft of WT or transfecting miR-211-5p mimics LM3 cells and detection of ACSL4 expression in vivo. **a** Representative images of tumors of the two groups in the BALB/c nude mice. **b** Comparison of tumor volume of the two groups every 5 days. **c** Comparison of tumor weight of the two groups 1 month after the subcutaneous injection. **d** Comparison of body weight of the two groups every 5 days. **e** The protein expression level of ACSL4 and β-Actin in 5 WT LM3 and 5 miR-211-5p mimics transfected LM3 xenograft tumors. **f** Representative immunohistochemistry images of WT and miR-211-5p mimics transfected LM3 xenograft tumor tissues with ACSL4 staining (×200). **g** Quantification analysis of H-score of ACSL4 staining in WT and miR-211-5p mimics transfected LM3 xenograft tumor tissues. *p < 0.05, **p < 0.01, ***p < 0.001

In addition to the investigation of the biological function of miR-211-5p in HCC cells in vitro, we also examined whether miR-211-5p modulates ACSL4 expression and tumorigenesis of HCC cells in vivo. By establishing a xenograft model in vivo by subcutaneous injection with WT LM3 cells or those transfected with miR-211-5p mimic, miR-211-5p overexpression clearly reduced the volume and weight of the xenograft tumors (Fig. 6a–c), although no apparent difference in body weight between the two groups (Fig. 6d) was observed. The expression of ACSL4 in WT LM3 tumors and those transfected with miR-211-5p mimic were then measured. As shown in Fig. 6e–g, miR-211-5p overexpression by mimics in xenograft tumors substantially suppressed the expression of ACSL4, further demonstrating that miR-211-5p inhibited ACSL4 expression in HCC. Together, the results indicate that miR-211-5p overexpression suppressed the expression of ACSL4 and impaired the tumorigenesis and growth of HCC in vivo.

## Discussion

MiRNAs are a group of non-coding RNA molecules with a length of 18–25 nucleotides which play an important role in the regulation of gene transcription [20, 21]. MiRNAs negatively regulate gene expression by pairing with the 3′ UTR of target mRNA transcripts leading to degradation or translational inhibition of the target mRNA [20, 22]. In recent years, increasing numbers of studies have confirmed that miRNA is closely related to tumor progression that can be used as a tumor oncogene, tumor suppressor gene or signaling molecule to participate in the regulation of tumor biological processes such as proliferation, apoptosis, invasion, metastasis, or angiogenesis [23–26]. The miR17-92 family, currently recognized as cancer-promoting miRNAs, are abnormally highly expressed in a variety of tumors [27]. Conversely, the miR-34 family, which targets p53, can indirectly inhibit the development of tumors [28].

MiR-211-5p has been shown to be down-regulated in osteosarcoma, promote apoptosis, and inhibit the migration of osteosarcoma cells by targeting the proline-rich protein PRR11 [29]. Likewise, Chen et al*.* also reported that the expression of miR-211-5p was low in triple-negative breast cancer, serving as a tumor suppressor in the development of breast cancer by targeting SETBP1 [30]. Thus, the important role of deregulated miR-211-5p in HCC has been established [31–33]. Jiang et al. [31] found that miR-211-5p expression is downregulated in HCC cells and tissues compared with corresponding non-tumor tissues. The in vitro experiments illustrate that overexpression of miR-211-5p inhibits the invasion of HCC cells whereas miR-211-5p inhibitor promotes cell invasion. The in vivo experiments demonstrate that the volumes and weights of tumors in mouse xenografts treated by miR-211-5p mimic are also lower than that observed in tumors treated with a scrambled mimic. Moreover, Jiang et al*.* confirmed that miR-211-5p was able to repress tumor formation in HCC by directly targeting STAB2. Furthermore, Deng et al. [32] discovered that low levels of miR-211-5p were associated with TNM stage, vein invasion status, and poor prognosis, with ectopic expression of miR-211-5p effective in suppression of cell proliferation, migration, and invasion both in vitro and in vivo by directly targeting SPARC. Jiang et al. [33] demonstrated that miR-211-5p suppressed HCC by inhibition of ZEB2 expression. All three studies reported that miR-211-5p was down-regulated in HCC and that miR-211-5p inhibited the progression of HCC by modulating different target genes, similar to the findings of the present study. However, no existing studies have elucidated the relationship between miR-211-5p and ACSL4 in HCC and the effect of ACSL4 overexpression on HCC cell lines. In the present study, we first proposed that enhanced expression of ACSL4 promoted a malignant phenotype in HCC cells. We then established that miR-211-5p suppressed the proliferation, migration, and invasion of HCC cells by negative regulation of ACSL4. Furthermore, we found that overexpression of miR-211-5p inhibited ACSL4 expression in a xenograft tumor model. In summary, these results demonstrated that miR-211-5p may act as a tumor suppressor in HCC by targeting ACSL4.

ACSL4, also described as FACL4, encodes a protein that is an isozyme of the long-chain fatty-acid-coenzyme A ligase family [16]. Its absence may contribute to cognitive disability or Alport syndrome [13]. In addition, the abnormal expression of ACSL4 is closely associated with diabetes [34], atherosclerosis [35], obesity [36], and a series of malignant tumors [14, 17, 18, 37–39]. Although it has been reported that ACSL4 is upregulated in HCC [17, 18]], the underlying mechanisms of ACSL4 overexpression in HCC and the effect of ACSL4 on the progression of HCC remain unclear. Interestingly, the present study demonstrated that ACSL4 was a direct downstream target of miR-211-5p in HCC and that miR-211-5p can negatively regulate the expression levels of ACSL4. Furthermore, ACSL4 was able to rescue HCC cell proliferation, invasion, and migration following miR-211-5p overexpression.

The present study identified miR-211-5p as the sole DEmiR and its downstream target as ACSL4 in HCC based on bioinformatics analysis and experimental validation. However, the research still had the following limitations that cannot be ignored: Firstly, the datasets used were obtained from the GEO database, not from RNA sequencing of patient samples, which may have been more reliable. Secondly, it was confirmed that miR-211-5p served as a tumor suppressor in HCC by targeting ACSL4, although the underlying mechanism of the promotion of cell proliferation, invasion, and migration by ACSL4, remains unclear. Thirdly, more detailed research is required to further elucidate the molecular mechanisms and signaling pathways through which miR-211-5p regulates ACSL4 expression in HCC.

## Conclusions

In conclusion, this study demonstrated that miR-211-5p was substantially down-regulated in HCC tissues and cell lines compared with normal tissue, whereas ACSL4 was up-regulated in HCC cell lines. Additionally, survival analysis indicated that HCC patients with decreased expression of miR-211-5p had poor overall survival. Importantly, miR-211-5p suppressed cell proliferation, migration, and invasion by inhibition of downstream ACSL4. Finally, we confirmed that miR-211-5p overexpression inhibited expression of ACSL4 and impaired the tumorigenesis and growth of HCC in vivo. Our findings indicate that targeting miR-211-5p and the downstream ACSL4 gene may provide novel insights and represents a promising approach for future therapy of HCC patients.

## Supplementary information

**Additional file 1: Figure S1.** The expression of miR-211-5p and ACSL4 in HCC cell lines. (A) LM3 and SK Hep-1 cells were both transfected with miR-211-5p mimics and inhibitor. The effect of transfection was tested by qRT-PCR: the miR-211-5p mimic expression in LM3 cells exceeded expression levels of SK Hep-1 WT cells, and the inhibitor declined expression of miR-211-5p in SK Hep-1 cells to comparable levels of LM3 WT cells. (B) The qRT-PCR and western blot showed that ACSL4 was up-regulated in 4 HCC cell lines (Huh-7, SK Hep-1, HepG2, and LM3) compared with normal liver cells (QSG-7701). *p<0.05, **p<0.01, ***p<0.001.

**Additional file 2: Figure S2. **Overexpression of ACSL4 facilitates proliferation, migration, and invasion of HCC cells. (A) The mRNA and protein expression level of ACSL4 in control group, transfecting siACSL4-1 group, transfecting siACSL4-2 group, and transfecting pcDNA3.1-ACSL4 group. (B) The proliferation ability of LM3 and SK Hep-1 cells in control group, transfecting siACSL4-2 group, and ACSL4 overexpression group. (C) Comparison of migration and invasion of LM3 cells between control group, siACSL4-2 group, and ACSL4 overexpression group. (D) Comparison of migration and invasion of SK Hep-1 cells between control group, siACSL4-2 group, and ACSL4 overexpression group. (E) Quantification analysis of results from (C). (F) Quantification analysis of results from (D). *p<0.05, **p<0.01, ***p<0.001.

## Data Availability

The datasets analyzed in this study available from the corresponding author on request.

## Abbreviations

HCC: Hepatocellular carcinoma
MiRNAs: MicroRNAs
GEO: Gene expression omnibus
KNN: k-nearest neighbor
TCGA: The Cancer Genome Atlas
DMEM: Dulbecco’s modified Eagle’s medium
FBS: Fetal bovine serum
NC: Negative control
DEmiRs: Differentially expressed miRNAs
MiR-211-5p: MicroRNA-211-5p
CCK-8: Cell counting kit-8 reagent
ACSL4: Acyl-CoA Synthetase Long Chain Family Member 4
siRNAs: Small interfering RNAs
WT: Wild-type
MUT: Mutant
IHC: Immunohistochemistry
BSA: Bovine serum albumin
OE: Overexpression
