# Supplementary figures and images for: Identification of MiR-211-5p as a tumor suppressor by targeting ACSL4 in Hepatocellular Carcinoma
Source: J Transl Med. 2020 Aug 28;18:326. doi: 10.1186/s12967-020-02494-7 (PMC7456023; doi:10.1186/s12967-020-02494-7)

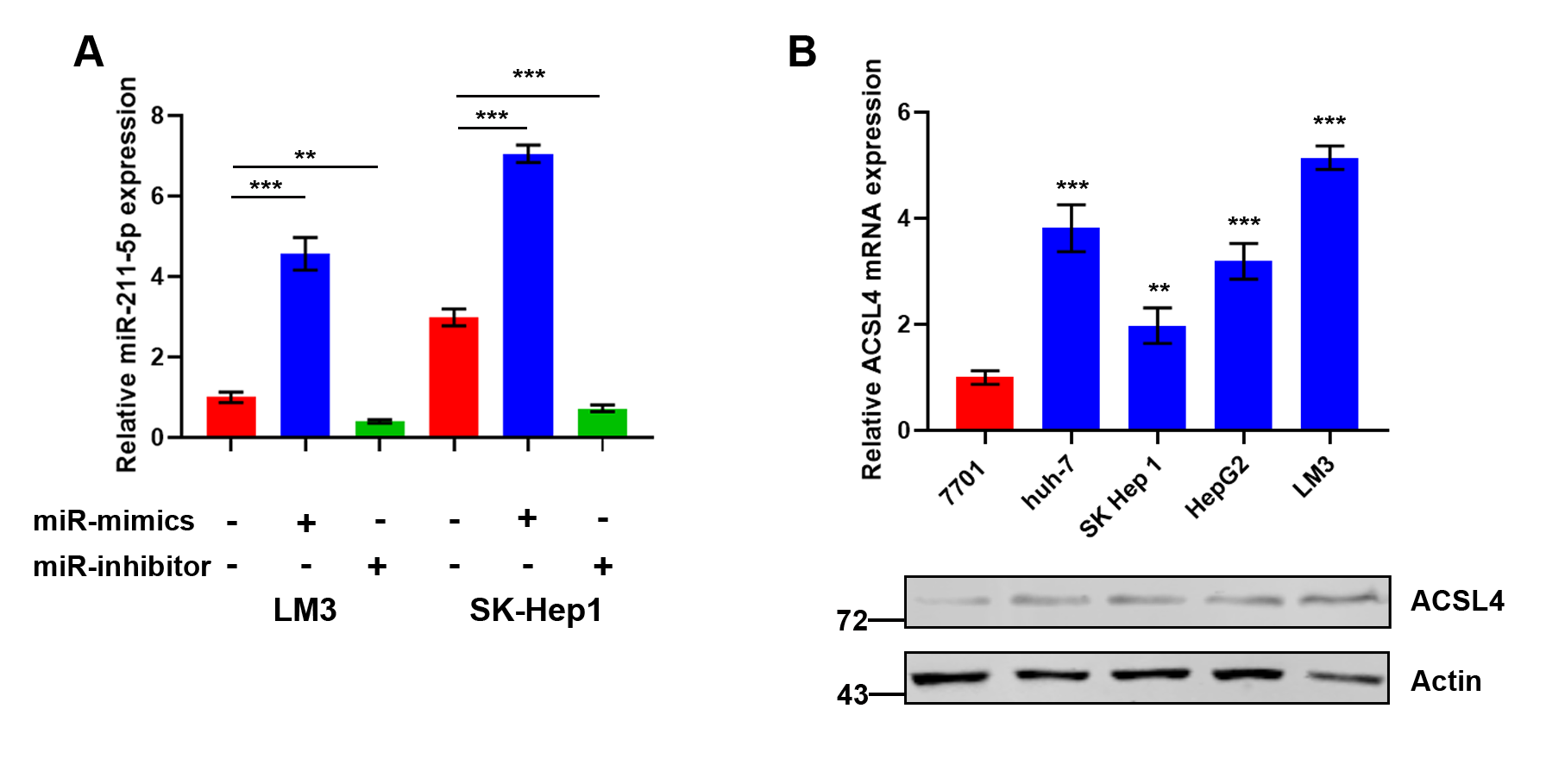

Supplement: Supplementary file 1 — Additional file 1: Figure S1. The expression of miR-211-5p and ACSL4 in HCC cell lines. (A) LM3 and SK Hep-1 cells were both transfected with miR-211-5p mimics and inhibitor. The effect of transfection was tested by qRT-PCR: the miR-211-5p mimic expression in LM3 cells exceeded expression levels of SK Hep-1 WT cells, and the inhibitor declined expression of miR-211-5p in SK Hep-1 cells to comparable levels of LM3 WT cells. (B) The qRT-PCR and western blot showed that ACSL4 was up-regulated in 4 HCC cell lines (Huh-7, SK Hep-1, HepG2, and LM3) compared with normal liver cells (QSG-7701). *p<0.05, **p<0.01, ***p<0.001. [file 12967_2020_2494_MOESM1_ESM.tif]

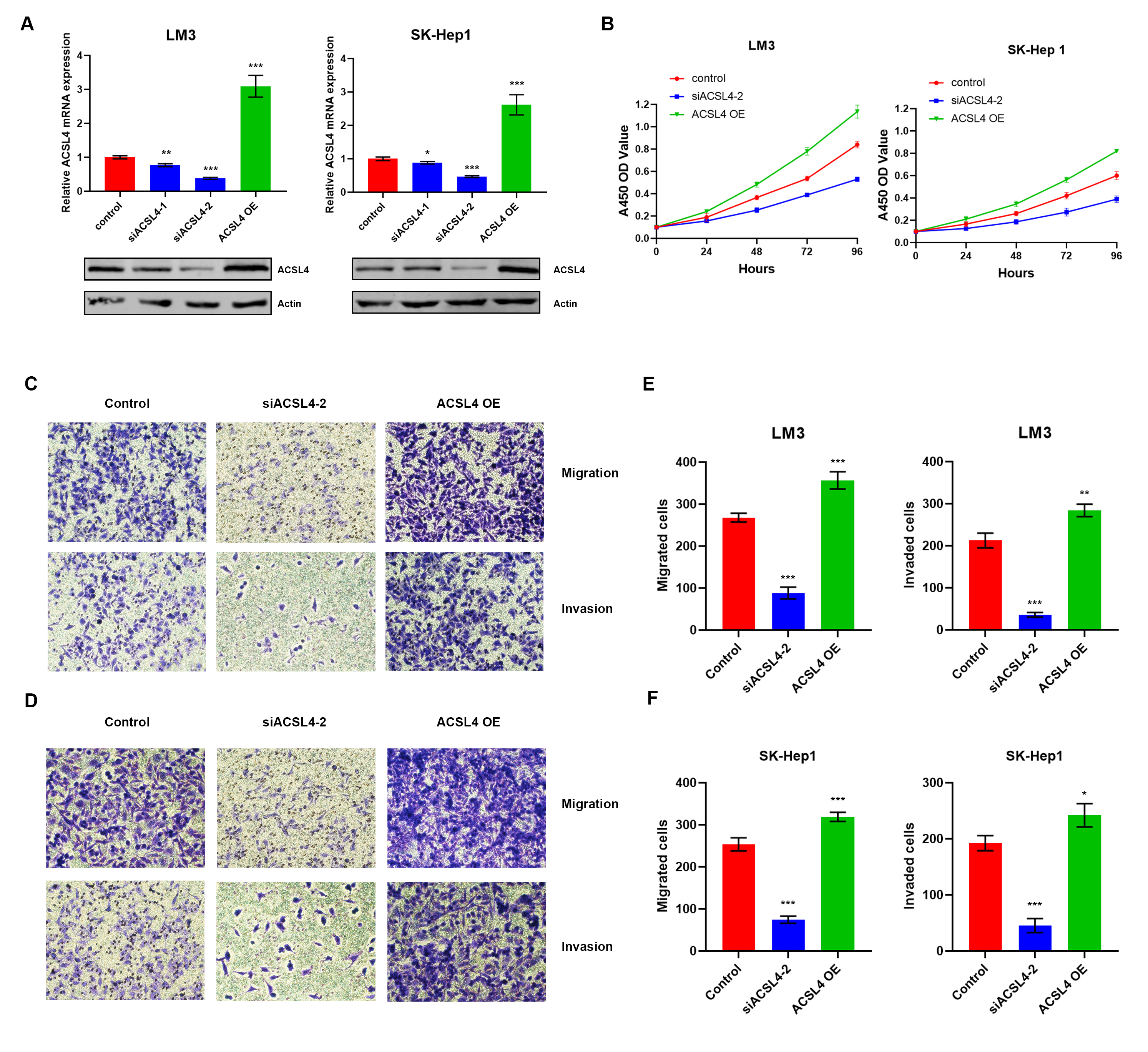

Supplement: Supplementary file 2 — Additional file 2: Figure S2. Overexpression of ACSL4 facilitates proliferation, migration, and invasion of HCC cells. (A) The mRNA and protein expression level of ACSL4 in control group, transfecting siACSL4-1 group, transfecting siACSL4-2 group, and transfecting pcDNA3.1-ACSL4 group. (B) The proliferation ability of LM3 and SK Hep-1 cells in control group, transfecting siACSL4-2 group, and ACSL4 overexpression group. (C) Comparison of migration and invasion of LM3 cells between control group, siACSL4-2 group, and ACSL4 overexpression group. (D) Comparison of migration and invasion of SK Hep-1 cells between control group, siACSL4-2 group, and ACSL4 overexpression group. (E) Quantification analysis of results from (C). (F) Quantification analysis of results from (D). *p<0.05, **p<0.01, ***p<0.001. [file 12967_2020_2494_MOESM2_ESM.jpg]
